# Validating the International Classification of Functioning, Disability and Health Core Sets for Autism in a Sample of Australian School-Aged Children on the Spectrum

**DOI:** 10.1007/s10803-024-06295-5

**Published:** 2024-02-24

**Authors:** Maya Hayden-Evans, Kiah Evans, Benjamin Milbourn, Emily D’Arcy, Angela Chamberlain, Bahareh Afsharnejad, Andrew Whitehouse, Sven Bölte, Sonya Girdler

**Affiliations:** 1 Curtin Autism Research Group, School of Allied Health, Curtin University, Perth, 6102 Australia; 2 Telethon Kids Institute, University of Western Australia, Perth, 6009 Australia; 3 Autism CRC, Long Pocket, Brisbane, QLD 4850 Australia; 4 School of Allied Health, University of Western Australia, Perth, 6009 Australia; 5 School of Psychological Science, University of Western Australia, Perth, 6009 Australia; 6 Center of Neurodevelopmental Disorders (KIND), Centre for Psychiatry Research, Department of Women’s and Children’s Health, Karolinska Institutet & Stockholm Health Care Services, Region Stockholm, Stockholm, 104 31 Sweden; 7 Child and Adolescent Psychiatry, Stockholm Health Care Services, Region Stockholm, Stockholm, 104 31 Sweden

**Keywords:** Assessment, Autism, Content Validity, Functioning, ICF core sets, Measure

## Abstract

Assessing functioning of children on the autism spectrum is necessary to determine the level of support they require to participate in everyday activities across contexts. The International Classification of Functioning, Disability and Health (ICF) is a comprehensive biopsychosocial framework recommended for classifying health-related functioning in a holistic manner, across the components of body functions, activities and participation, and environmental factors. The ICF Core Sets (ICF-CSs) are sub-sets of relevant codes from the broader framework that provide a basis for developing condition-specific measures. This study combined the ICF-CSs for autism, attention deficit hyperactivity disorder (ADHD) and cerebral palsy (CP) to validate the ICF-CSs for autism in an Australian sample of school-aged children. This cross-sectional study involved caregivers of school-aged children on the spectrum (*n* = 70) completing an online survey and being visited in their homes by an occupational therapist to complete the proxy-report measure based on the ICF-CSs for autism, ADHD and CP. Absolute and relative frequencies of ratings for each of the codes included in the measure were calculated and reported, along with the number of participants who required clarification to understand the terminology used. Findings indicate that the body functions and activities and participation represented in the ICF-CSs for autism were the most applicable for the sample. However, findings relating to environmental factors were less conclusive. Some codes not currently included in the ICF-CSs for autism may warrant further investigation, and the language used in measures based on the ICF-CSs should be revised to ensure clarity.

## Introduction

The concept of functioning is complex and multi-faceted, involving interaction between multiple factors, including both internal and external influences on an individual’s ability to participate in daily life (World Health Organisation [WHO], 2013). Children on the autism spectrum experience varying degrees of difference in social communication and social interactions, and engage in repetitive patterns of behaviour, interests or activities that can impact their functioning across a variety of contexts (American Psychiatric Association [APA], 2022). In Australia, an assessment of functioning is recommended before or during the autism diagnostic process to identify barriers and facilitators to participation, and guide the allocation of supports (Goodall et al., 2023; Whitehouse et al., 2018). It is recommended that this process utilise multiple methods of gathering information, including parent interview, clinical observations, and use of standardised measures, to obtain a comprehensive overview of the child’s functioning (Whitehouse et al., 2018). Use of the International Classification of Functioning, Disability and Health (ICF) and associated ICF Core Sets (ICF-CSs) is recommended to guide the process of obtaining a strengths-focused and holistic overview of the person, as opposed to focusing only on the diagnostic criteria (Whitehouse et al., 2018). Thus, the current study aimed to validate the ICF-CSs for autism in an Australian context with a focus on school-aged children, given the large prevalence of autism in this age group (Australian Bureau of Statistics, 2019).

Currently, the average age at autism diagnosis is approximately 5 years (van’t Hof et al., 2021). However, the age at which a diagnosis is received can vary depending on a variety of factors, including clarity of clinical features, sociodemographic factors, level of parental concern, access to services, geographic location, and cohort effects (Daniels & Mandell, 2013). Characteristics of autism in school-aged children, generally considered between the ages of 6 and 16 years (Services Australia, 2022), may have been overlooked during early childhood, and become more apparent during the school years, when the expectations placed upon the child begin to exceed their capabilities (Avlund et al., 2021). During this period, children experience increased social demands, strive towards independence in a variety of daily living situations, and are expected to function effectively across multiple contexts (Avlund et al., 2021). The school-aged years represent a critical period of social development, when children establish friendships, self-esteem, and personal identity, and begin to understand societal expectations for behaviour during social situations (Kwon et al., 2014). Measures assessing these critical areas of functioning can be used to determine a child’s abilities relative to their same-aged peers, as well as identify areas of strength and difficulty, and provide an initial point of reference against which progress can be measured (Whitehouse et al., 2018). However, the scope of existing measures of functioning for school-aged children on the spectrum is limited (Hayden-Evans et al., 2022).

The ICF is the WHO’s biopsychosocial framework for classifying and describing health-related functioning (WHO, 2013). The ICF consists of over 1600 codes designed to comprehensively capture aspects of functioning across the components of body functions (physiological functions of body systems) and structures (anatomical body parts), activities (task or action carried out by a person), participation (involvement in life situations), and environmental (physical, social, attitudinal) factors. To improve the utility of the ICF in research and clinical settings, shortlists of ICF codes most relevant to particular conditions have been developed, called the ICF Core Sets (ICF-CSs; Selb et al., 2015). These have been established for a range of conditions, including autism (Bölte et al., 2019), attention deficit hyperactivity disorder (ADHD; Bölte et al., 2018) and cerebral palsy (CP; Schiariti et al., 2015), following a rigorous process endorsed by the WHO (Selb et al., 2015). In their original form, the ICF-CSs provide a standard for describing the areas of functioning most relevant to individuals with a particular condition and can be used to guide the development of condition-specific measures of functioning (Selb et al., 2015).

In Australia, a transdiagnostic approach to assessing functioning is recommended when an autism diagnosis is being considered (Whitehouse et al., 2018). Neurodevelopmental conditions (NDCs) typically present during the early developmental period and involve impairment across multiple areas of functioning due to differences in the brain’s processing abilities (APA, 2022). Included in the Diagnostic and Statistical Manual of Mental Disorders (DSM-5-TR; APA, 2022) and International Classification of Diseases (ICD-11; WHO, 2019), the two most prevalent NDCs in children are autism and ADHD (Scandurra et al., 2019). Although not included in the DSM-5-TR (APA, 2022), and classified as a disease of the nervous system under the ICD-11 (WHO, 2019), CP is also considered a neurodevelopmental condition (Schiariti et al., 2018; The Lancet, 2013) and is the most common cause of motor disability in children (Centers for Disease Control and Prevention, 2022). Features of autism often overlap with those of other NDCs and it is common for an individual with one NDC to have co-occurring NDCs (Hansen et al., 2018), making it difficult to determine which characteristics are attributable to each condition.

Given the complex nature of autism and the high prevalence of co-occurring neurodevelopmental conditions, it is important to ensure that autism-specific measures do in fact assess the specific features of autism and are not confounded by the characteristics of other, similar conditions. Content validity, referring to how accurately the content of a measure reflects what it intends to assess (Mokkink et al., 2012), is a crucial yet often underrated and under-evaluated step in the process of developing measures that should be completed prior to evaluation of other psychometric properties (Terwee et al., 2018). Establishing content validity is a purely judgemental process, involving the following steps: (1) considering construct information; (2) considering content of the measure; (3) selecting a panel of experts; (4) evaluating the content of the measure for relevance and comprehensiveness; and (5) using a framework to evaluate the relationship between the measure and construct (Terwee et al., 2011). These steps align with those in developing ICF-CSs, requiring a series of preparatory studies including a systematic review of the literature, expert survey, and qualitative study prior to a consensus process, during which decisions are made about which ICF codes should be included (Selb et al., 2015). It is recommended that the ICF-CSs be implemented in practice for further evaluation following the consensus conference, however, during the development of the ICF-CSs for autism, a clinical cross-sectional study was conducted prior to the consensus, with the findings used to inform the decision-making process (Mahdi et al., 2018).

The operationalisation of multiple ICF-CSs has been achieved through the development of condition-specific outcome measures based on the relevant codes included in ICF-CSs (Sengers et al., 2021; van Leeuwen et al., 2020; Yang et al., 2014). However, no such measure has so far been developed to assess functioning of school-aged children on the spectrum. Other measures used to capture aspects of functioning in this population have previously been mapped to the ICF and associated ICF-CSs for autism, finding limited representation of body functions and environmental factors relative to activities and participation (Hayden-Evans et al., 2022). Using the ICF-CSs as the basis for new, condition-specific measures provides a starting point for determining which items should be included in the measure. However, it is important when developing measures to establish face validity by considering how easily understood the measure will be by the target population, including consideration of ambiguous language and excessive use of jargon (Streiner et al., 2015). While the ICF strives to provide a common language for ease of communication between clinicians, researchers and policy makers (WHO, 2013), the suitability of this language for use in patient- or proxy-reported measures designed to be completed by the general population has not yet been established.

This study utilised a preliminary version of a measure based on the combined codes of the ICF-CSs for autism, ADHD and CP to further evaluate the finalised ICF-CSs for autism and determine their content validity via hypothesis testing in an Australian context. We hypothesised that, in a sample of school-aged children on the spectrum, the challenges, barriers and supports identified by proxy-reporting caregivers would correspond to codes included in the ICF-CSs for autism. In order to investigate this hypothesis, the following objectives were identified:

1. Explore the frequency at which caregivers indicated their child on the spectrum experienced impairment, difficulty, barriers, and facilitators in areas relevant to the codes included in the combined ICF-CS for NDCs.
2. Identify whether the codes included in the ICF-CSs for autism were the most applicable for a sample of Australian school-aged children on the spectrum.
3. Determine caregivers’ understanding of the operational definitions of the codes included in the ICF-CS for NDCs.

## Materials and Methods

### Study Design

This national, cross-sectional study was embedded within a larger program of research that sought to evaluate the psychometric properties of measures of functioning and evaluate assessment of functioning processes in an NDC context (Evans et al., 2022). The focus of this study was on confirming the content validity of the ICF-CSs for autism, building on previous studies that informed their development (Bölte et al., 2019; Mahdi et al., 2018). This study involved caregivers of children on the spectrum rating their child’s functioning, including the impact of environmental factors, using a preliminary version of a measure based on the combined ICF-CSs for NDCs. A cross-sectional design was selected to enable comparison of functioning in a sample of school-aged children on the spectrum at a single point in time (Portney & Watkins, 2013).

### Participants

Convenience sampling, whereby participants are selected based on eligibility and availability (Portney & Watkins, 2013), was used to recruit participants for the broader program of research who were caregivers of an individual under the age of 21 with a NDC diagnosis and registered with the National Disability Insurance Scheme (NDIS), living in one of the following Australian states: New South Wales, Queensland, Victoria or Western Australia. Researchers shared information about the research program with their networks and promoted it on social media. The National Disability Insurance Agency (NDIA) assisted with recruitment by inviting eligible caregivers of children and young people registered with the NDIS to participate. Participation in the study was entirely voluntary and families who did not wish to participate in the research declined the invitation without consequence. Data from a sub-sample of 70 caregivers reporting on 67 children on the spectrum were determined eligible for inclusion in the analysis for this study. These participants were caregivers of a school-aged child, between 6 and 16 years, with an autism diagnosis, who also met the aforementioned inclusion criteria for the broader program.

### Measures

Participants provided sociodemographic information via an online survey, which included questions about both the caregiver/s and their child on the spectrum. A series of other, established measures were utilised to document observed features of autism and provide a description of functioning.

#### Autism Mental Status Examination (AMSE)

The AMSE is a short observational measure providing clinicians with a guide for observing and documenting features of autism during an interaction with a child on the spectrum (Grodberg et al., 2012). Each item (eye contact, interest in others, pointing, language, pragmatics, repetitive behaviours, preoccupations and sensitivities) is scored 0, 1, or 2, with the scoring criteria for each individual item described in the user manual. The AMSE has demonstrated excellent sensitivity and specificity in both adults (Grodberg et al., 2015) and children (Galdino et al., 2020), with one study using a relevant sample of children and adolescents finding a sensitivity of 0.91 and specificity of 0.98 with a cutoff of 4 points (Galdino et al., 2020).

#### Pediatric Evaluation of Disability Inventory Computer Adaptive Test (PEDI-CAT)

The PEDI-CAT is a proxy-report, computerised adaptive test measuring the abilities of children and young people up to the age of 20 years in the domains of daily activities, mobility and social/cognitive, as well as a child’s level of responsibility in managing their own life tasks (Haley et al., 2012). A version of the PEDI-CAT has been validated for children and young people with a diagnosis of ASD, including new or revised items as well as further directions for rating items, with consideration of the specific features of children on the spectrum (Haley et al., 2018). The PEDI-CAT (ASD) was utilised in this study. For the PEDI-CAT (ASD), normative scores are presented as T-scores, where the mean for each age group is 50 and the standard deviation is 10 (Haley et al., 2018). Scores between 30 and 70, or within two standard deviations of the mean, are regarded as within the typical range expected for that age group (Haley et al., 2018).

#### Vineland Adaptive Behavior Scales, Third Edition (Vineland-3)

The Vineland-3 (Sparrow et al., 2016) measures adaptive behaviour of people with a variety of disabilities and is suitable for use across the lifespan. There are multiple Vineland-3 forms relevant to specific ages and informants; in this study, the caregiver interview form was used. This measure has strong psychometric properties, with internal consistency ranging from 0.90 to 0.98 and interrater reliability from 0.70 to 0.81 for the comprehensive interview form (Hill et al., 2021). The Vineland-3 covers four domains of adaptive functioning: (1) communication; (2) daily living skills; (3) socialisation; and (4) motor skills, and includes an optional scale of maladaptive behaviours. During the interview, caregivers are prompted to describe their child’s behaviour across each of the domains and the interviewer determines whether the child ‘usually/regularly’, ‘sometimes’ or ‘does not’ demonstrate the behaviour (Sparrow et al., 2016). For the Vineland-3, normative scores are presented as standard scores, where the mean is 100 and the standard deviation is 15 (Sparrow et al., 2016).

#### Prototype Proxy-Report Measure Based on the ICF-CSs for NDCs (ICF-NDCs)

A prototype proxy-report measure of functioning based on the ICF-CSs for autism (Bölte et al., 2019), ADHD (Bölte et al., 2018) and CP (Schiariti et al., 2015) was developed for use in this study and piloted with caregivers (*n* = 10) prior to data collection for this study. The measure consists of 161 s- and third-level codes from the components of body functions (BF; 44 codes), activity and participation (AP; 76 codes), and environmental factors (EF; 41 codes). Body structures were excluded as these refer to anatomical body parts including organs and limbs (WHO, 2013), which were considered inappropriate for inclusion in a proxy-report measure given the need for medical evaluation to accurately assess these. Each code was rated on a modified five-point scale, based on the ICF qualifiers, which defines level of functional impairment (BF), difficulty (AP) or barrier (EF) according to how often a problem presents: 0, No problem (0–4%); 1, Mild problem (5–24%); 2, Moderate problem (25–49%); 3, Substantial problem (50–95%); 4, Complete problem (96–100%; WHO, 2013). In the ICF, EF are considered along a scale of opposing poles, with barriers at one end and facilitators at the other (WHO, 2013). Following the pilot, adjustments were made to the scales for rating EF to enable participants to rate each code according to how much of a barrier and a facilitator it was for their child. This change was made to capture the possibility of EF operating as two independent constructs, with each factor having the potential to support or impede functioning depending on the context (Anaby et al., 2013).

**Fig. 1:**
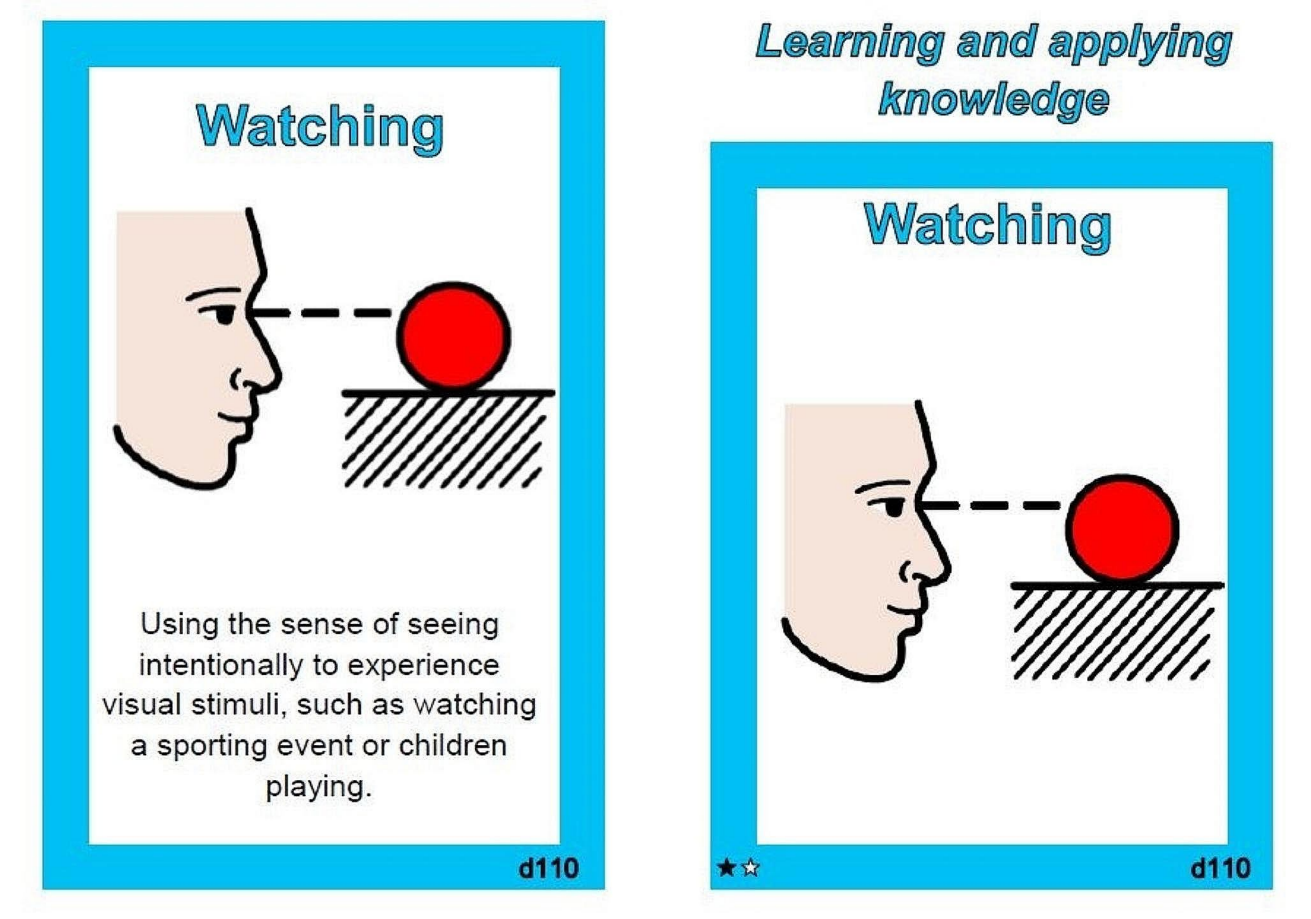
Example of a card presented to participants during administration of the ICF-NDCs

To improve the utility of the ICF-CSs for NDCs, researchers adapted the format of the ICF-CS documentation form available to download online (ICF Research Branch, 2023). Caregivers were provided with a deck of 161 cards, each containing the title of a code from the combined ICF-CSs for NDCs, a diagram visually representing the code, and the complete definition of that code (copied verbatim from the ICF-CS documentation form) on the reverse side of the card (see Fig. 1). A set of modified definitions developed by the occupational therapists in the research team were available to aid understanding if required.

Caregivers were presented with a physical scale on which to place each card and an assessor recorded their responses in an online version of the ICF-NDCs. Participants were able to seek clarification from the assessor regarding any uncertainties about the codes, for example, if they did not understand what the code meant, however, the assessor did not provide guidance around where to place the card. The assessor recorded each time a participant requested clarification of a code, to help determine caregivers’ understanding of the current definitions.

### Data Collection

This study was approved by Bellberry Ethics (HREC approval 2018-10-852), with reciprocal approval granted by Curtin University Human Research Ethics Committee (HREC approval HRE2019-0001). Potential participants completed an online survey determining their eligibility to be included in the study. Following this, eligible participants were provided with a link to another online survey containing further information about the study and consent. All participants were required to provide informed consent via the online survey prior to continuing with the study, which involved the completion of a sociodemographic questionnaire (Evans et al., 2022), followed by a home visit from a researcher who was also a registered occupational therapist to complete the ICF-NDCs, as well as the PEDI-CAT (ASD), which the caregiver completed on a computer during the home visit. Researchers recorded the caregivers’ responses to the ICF-NDCs in an online form hosted on data management platform, RedCap (Harris et al., 2019), along with their own responses to the items of the AMSE following interaction with the child. Participants were contacted by a different occupational therapist to complete the Vineland-3 interview via telephone.

### Data Analysis

Participant demographic information was descriptively analysed and is summarised and reported in Table 1. Absolute (*n*) and relative (%) frequencies of ratings and clarification required for each of the codes included in the components of the ICF-NDCs were calculated and are reported in Appendices B-D. Additionally, absolute and relative frequencies of participants who rated a code as ≥ 2 were combined and reported to identify the number of participants who considered each code to be at least a moderate problem, excluding those who were unable to rate the code or considered the code not applicable. A cut-off of ≥ 2 was selected as, according to the ICF, a moderate problem is one that occurs up to half of the time or represents half the scale of total difficulty (WHO, 2013). Mild problems, occurring between 5% and 24% of the time, are less likely to significantly impact functioning and were therefore excluded, in keeping with a previous study of functioning using the ICF-CS for autism (Mahdi et al., 2018).

**Table 1:** Caregiver and child sociodemographic information

Variable	n (%)
**Caregiver gender**	
Male	3 (4)
Female	67 (96)
Other	0 (0)
**Caregiver age**	
Range	29–67 years
Mean	42.68 years
SD	7.12 years
**Relationship to child**	
Biological parent	69 (99)
Grandparent	1 (1)
**State**	
New South Wales	14 (20)
Queensland	6 (9)
Victoria	29 (41)
Western Australia	21 (30)
**Child gender**	
Male	42 (63)
Female	25 (37)
Other	0 (0)
**Child age**	
Range	6–16 years
Mean	10.61 years
SD	2.88 years
**Primary and co-occurring conditions**	
Autism spectrum disorder (DSM-5)	56 (84)
Autistic disorder (DSM-IV)	8 (12)
Asperger’s syndrome	8 (12)
Pervasive developmental disorder – not otherwise specified	5 (7)
Attention deficit hyperactivity disorder	25 (37)
Communication, language or speech disorder	14 (21)
Coordination, motor or movement disorder	9 (13)
Cerebral palsy	0 (0)
Global developmental delay	6 (9)
Intellectual disability	9 (13)
Learning disorder	4 (6)
Foetal alcohol spectrum disorder	0 (0)
Tic disorder	3 (4)
Other	9 (13)
**AMSE score**	
Range	0–10
Mean	5.06
SD	2.39
**Age at diagnosis**	
Range	1–13 years
Mean	5.79 years
SD	3.02 years

## Results

### Sociodemographic and Clinical Results

A total of 70 caregivers completed the ICF-NDCs for 67 school-aged children on the spectrum. Caregivers were predominantly mothers (96%). Three sets of parents completed the measure together, with both the mothers and fathers reporting on their child’s functioning, and one grandparent completed the measure. There were more male (63%) than female (37%) children reported on. The majority of children had a diagnosis of ASD, with some reporting an earlier diagnostic label under previous versions of the DSM. The most common co-occurring NDC was ADHD (37%). The average AMSE score calculated for this sample was 5.06; a score of ≥ 5 has been found to predict autism with 94% sensitivity and 81% specificity (Grodberg et al., 2012). Further participant sociodemographic information is provided in Table 1.

All children scored below the normative mean of 100 on the Vineland-3 for overall level of adaptive functioning, with an average adaptive behaviour composite of 66.35 (SD = 13.16). The T-scores across domains of functioning of the PEDI-CAT (ASD) indicate the sample performed, on average, below age expectations in daily activities (M = 28.93). The average T-scores across other domains of functioning reflected performance within the lower range expected for their age. The range, mean and standard deviation of Vineland-3 standard scores and PEDI-CAT (ASD) T-scores for the sample of children on the spectrum included in this study are reported in Table 2.

**Table 2:** Functioning of school-aged children on the autism spectrum measured using the Vineland-3 and PEDI-CAT (ASD)

Vineland-3 standard scores					
	Adaptive behaviour composite	Daily living skills	Motor	Communication	Socialisation
Range	23–85	20–114	20–109	20–102	24–90
Mean	66.35	72.64	77.67	65.24	63.06
SD	13.16	18.06	20.95	17.74	17.15
PEDI-CAT (ASD) T-scores					
		Daily activities	Mobility	Social/cognitive	Responsibility
Range		< 10–52	< 10–71	< 10–47	< 10–51
Mean		28.93	31.97	30.67	36.34
SD		11.21	15.61	9.51	8.15

### Caregiver Ratings of Functioning in School-Aged Children on the Spectrum Using the ICF-NDCs

#### Body Functions

The level of impairment of BF reported by caregivers in their school-aged children on the spectrum is reported in Appendix B. More than 50% of participants reported moderate to complete impairment in 11 of the 20 (55%) BF included in the ICF-CSs for autism, with more than 75% of participants reporting at least moderate impairment in the following BF codes: *b122 Global psychosocial functions (56, 84%), b125 Dispositions and intra-personal functions (54, 81%), b126 Temperament and personality functions (51, 76%), b140 Attention functions (59, 88%)*, and *b164 Higher-level cognitive functions (58, 87%)*. Between 18% (12) and 88% (59) of participants reported at least moderate impairment in the BF codes included in the comprehensive ICF-CS for autism (M = 23.30, SD = 16.49). Similarly, 19% (13) to 88% (59) of participants reported moderate to complete impairment in the BF codes included in the school-aged ICF-CS for autism (M = 23.62, SD = 16.79). Between 3% (2) and 55% (37) of participants reported at least moderate impairment in the BF codes not included in the ICF-CSs for autism (M = 13.04, SD = 9.03).

Mental functions were the most frequently reported impairments. A total of 55% (37) of participants reported that their school-aged child had moderate to substantial impairment in *b1301 Motivation*, which is not specifically included in the ICF-CSs for autism, but rather a third-level code that exists under *b130 Energy and drive functions*. More than half of participants (36, 54%) reported at least moderate impairment in *b163 Basic cognitive functions*, which is not included in the ICF-CSs for autism. Almost half of all participants reported no impairment in *b755 Involuntary movement functions* (35, 52%) and *b760 Control of voluntary movement functions* (33, 49%).

### Activity and Participation

The level of difficulty experienced by school-aged children on the spectrum in AP, as reported by their caregivers, is reported in Appendix C. At least half of all participants reported moderate to complete difficulty in 21 of the 59 (36%) AP codes included in the comprehensive ICF-CS for autism. The greatest areas of difficulty reported, indicated by majority rating of at least moderate difficulty, included: *d160 Focusing attention* (52, 78%), *d175 Solving problems* (52, 78%), *d220 Undertaking multiple tasks* (53, 79%), *d240 Handling stress and other psychological demands* (58, 87%), and *d720 Complex interpersonal interactions* (57, 85%), all of which are included in the school-aged ICF-CS for autism. Between 0% (0) and 87% (58) of participants reported moderate to complete difficulty in AP codes included in the comprehensive ICF-CS for autism (M = 23.34, SD = 15.91). For codes included in the school-aged ICF-CS for autism, between 16% (24) and 87% (58) of participants reported at least moderate difficulty (M = 26.35, SD = 16.35). In comparison, 3% (2) to 37% (25) of participants reported moderate to complete difficulty in AP codes not included in the ICF-CSs for autism (M = 9.71, SD = 7.95).

The greatest difficulty reported in AP was across the ICF chapters of: Learning and applying knowledge, General tasks and demands, Communication, and Interpersonal interactions and relationships. More than half of participants reported that their child had difficulty with *d166 Reading* (34, 51%) and *d170 Writing* (39, 58%), both of which are included in the comprehensive ICF-CS for autism but not the school-aged ICF-CS for autism.

### Environmental Factors

The level to which caregivers perceived EF to act as facilitators and barriers for their school-aged child on the spectrum is reported in Appendix D. There were more EF in the comprehensive ICF-CS for autism that were rated by ≥ 50% of participants as being at least moderate facilitators (27, 87%) than barriers (11, 35%). The EF included in the comprehensive ICF-CS for autism were rated as at least moderate facilitators by 13% (19) to 99% (66) of participants (M = 44.80, SD = 12.93). For EF included in the school-aged ICF-CS for autism, the range was the same (M = 45.46, SD = 12.56). For codes not included in the ICF-CSs for autism, between 22% (15) and 79% (53) of participants considered these EF to be at least moderate facilitators for their child (M = 38.10, SD = 10.30).

Almost all participants (66, 99%) reported that *e310 Immediate family* was at least a moderate facilitator for their child. Other EF reported to be at least a moderate facilitator by the majority of participants included: *e110 Products and substances for personal consumption* (51, 76%), *e115 Products and technology for personal use in daily living* (61, 91*%), e130 Products and technology for education* (63, 94%), *e250 Sound* (51, 76%), *e320 Friends* (54, 81%), *e330 People in positions of authority* (59, 88%), *e355 Health professionals* (60, 90%), *e360 Other professionals* (53, 79%), *e410 Individual attitudes of immediate family members* (58, 87%), *e420 Individual attitudes of friends* (50, 75%), *e430 Individual attitudes of people in positions of authority* (55, 82%), *e580 Health services, systems and policies* (57, 85%), and *e585 Education, training services, systems and policies* (54, 81%). The code, *e165 Assets*, was reported to be at least a moderate facilitator by 79% (53) of participants, although this code is not included in the ICF-CSs for autism.

Between 13% (19) and 78% (52) of participants considered EF included in the comprehensive ICF-CS (M = 26.78, SD = 11.61) and school-aged ICF-CS (M = 27.16, SD = 11.74) for autism to be at least moderate barriers. Between 4% (3) and 60% (40) of participants considered the EF not included in the ICF-CSs for autism to be at least moderate barriers for their child (M = 18.90, SD = 9.86). Environmental factors reported to be at least a moderate barrier by the majority of participants included *e250 Sound* (52, 78%) and *e460 Societal attitudes* (50, 75%).

### Codes Considered Not Applicable to Group

Ratings of not applicable (N/A) across the components of body functions, activity and participation, and environmental factors ranged from 0 to 100% (M = 12, SD = 23). Of the 161 codes included in the combined ICF-CSs for NDCs, 12 (7%) were considered N/A by more than half of all participants, with nine (6%) considered N/A by more than 75% of participants. Seven of the codes rated N/A by the majority of participants were from the AP component: *d770 Intimate relationships (90%), d815 Preschool education (88%), d825 Vocational training (99%), d830 Higher education (97%), d845 Acquiring, keeping and terminating a job (100%), d850 Remunerative employment (99%)*, and *d870 Economic self-sufficiency (82%).* The remaining two were EF: *e525 Housing services, systems and policies (75%)* and *e590 Labour and employment services, systems and policies (82%).*

Of these N/A codes, 10 are included in the comprehensive ICF-CS for autism, two are not included in the ICF-CSs for autism, and only *e590 Labour and employment services, systems and policies* is included in the school-aged ICF-CS for autism and not endorsed by participants in this study. None of the BF codes included in the ICF-NDCs were considered N/A by at least 50% of participants.

### Participant Understanding of Codes

Understanding of codes included in the ICF-NDCs varied, with between 0% and 55% (M = 19, SD = 12) of participants requiring further explanation of codes. Between 3% and 54% (M = 28, SD = 13) of participants required further explanation of BF codes. More than half of all participants requested further explanation of *b147 Psychomotor functions* (51%) and *b160 Thought functions* (54%). Between 0% and 25% (M = 12, SD = 7) of participants required further explanation of AP codes. The AP codes most frequently requiring further explanation were *d110 Watching* and *d137 Acquiring concepts*, both requested by 25% of participants. Between 3% and 55% (M = 22, SD = 10) of participants required further explanation of EF codes. The code, *e110 Products and substances for personal consumption*, required further explanation by the highest percentage of participants (55%).

## Discussion

This study aimed to measure functioning of school-aged children on the spectrum from their caregivers’ perspectives, using the ICF-NDCs, to further confirm the content validity of the ICF-CSs for autism. As expected, the BF and AP codes included in the ICF-CSs for autism were the most applicable for a sample of Australian school-aged children on the spectrum. However, findings relating to the EF codes were less conclusive. Across the components of BF and AP, the most significant areas of challenge for the sample were captured by the ICF-CSs for autism, with only one of the codes rated at least a moderate problem by more than half of participants corresponding with a second-level code from the ICF-CSs for ADHD or CP. However, there were multiple environmental factors rated as at least a moderate facilitator by more than half of participants that did not correspond with a code included in the ICF-CSs for autism. This finding suggests that in relation to environmental factors, and particularly facilitators, there may be significant overlap between autism, ADHD and CP. This supports a trans-diagnostic and individualised approach to assessing functioning of individuals with NDCs (Evans et al., 2022; Goodall et al., 2023; Whitehouse et al., 2018). The condition-specific ICF-CSs are necessary for capturing unique functional information, but may also be used flexibly with the option to add codes from the broader framework that may be relevant for a particular child or family (Schiariti et al., 2018).

### Body Functions

Regarding body functions, the findings of this study suggest that key areas of impairment for Australian children on the spectrum are mental functions encompassing psychosocial, cognitive, and attention abilities (*b122, b125, b126, b140 and b164).* Unsurprisingly, these codes correspond with core and associated features of autism outlined in the diagnostic criteria for ASD (APA, 2022; WHO, 2011). However, only 30% of participants reported moderate or greater impairment in involuntary movement functions (*b765*), despite the presence of repetitive behaviours including mannerisms and stereotypies being considered a core feature of autism. This finding suggests that the presence of these behaviours may not necessarily impair the child’s ability to function in daily life, supported by the findings of other studies, which found repetitive behaviours in autism can often serve to soothe the person and aid self-regulation (Collis et al., 2022; Kapp et al., 2019). Furthermore, this highlights that assessment of functioning and diagnostic evaluation are two related, but independent, clinical processes.

Drawing from the ICF-CS for CP, the codes *b1301 Motivation functions* and *b163 Basic cognitive functions*, were included in the ICF-NDCs and rated by more than half of caregivers as at least a moderate problem for their child. Motivation, not specifically included in the ICF-CSs for autism, exists under *b130 Energy and drive functions*, which is included, and encompasses motivation and related functions. These findings suggest that motivation may be a significant component of energy and drive issues in school-aged children on the spectrum, aligning with the findings of a previous study demonstrating lack of motivation, particularly regarding social interactions, can be a contributing factor in greater social skill impairment (Itskovich et al., 2021). While parents may consider issues with motivation in their rating of other energy and drive functions, emphasising the inclusion of motivation under this code may be necessary to ensure issues in this area are not missed. In addition to social situations, motivation is necessary for academic performance, and improving understanding of motivation issues in children on the spectrum may enable implementation of more targeted supports (Koegel et al., 2010).

Basic cognitive functions refer to those required for acquiring, organising and applying knowledge about objects, events and experiences (WHO, 2007). Despite relatively low prevalence of co-occurring intellectual disability in the sample, more than half of participants reported their child had at least moderate difficulty with basic cognitive functions. Autism is more often associated with higher level cognitive dysfunction, in such functions as organisation and planning, time management, and mental flexibility (Craig et al., 2016; Dijkhuis et al., 2020). Further investigation is required to determine whether these children really did demonstrate significant levels of impairment in basic cognitive functions, or if these results reflect parents’ level of understanding and perception of the difference between basic- and higher-level cognitive functions using the current definitions.

### Activity and Participation

The key difficulties identified for Australian children on the spectrum were from the AP chapters of Learning and applying knowledge (*d160* and *d175*), General tasks and demands (*d220* and *d240*), and Interpersonal interactions and relationships (*d720*). As expected, these key areas of difficulty correspond with codes included in both the comprehensive and school-aged ICF-CSs for autism. These codes reflect an array of activities required for success in school, incorporating both the academic and social challenges that may present during this period. Despite difficulties in social communication being among the core diagnostic criteria for autism (APA, 2022), this was not reflected in the findings of the current study. However, all children reported on in this study had an existing NDIS plan, enabling them access to services and supports, which may have contributed to improved social communication outcomes during the school-aged years (Fuller & Kaiser, 2020).

In previous iterations of the DSM, a diagnosis of autism excluded the possibility of ADHD, with any attentional difficulties being attributed to autism. However, in the most recent version of the DSM, it is acknowledged that the two conditions can, and often do, co-exist (APA, 2022; Scandurra et al., 2019). A total of 37% of the children on the spectrum included in this study had a co-occurring diagnosis of ADHD, which may have contributed to the significant difficulties reported in focusing and directing attention. However, even in children on the spectrum without a co-occurring ADHD diagnosis, difficulties with attention are common (Spaniol et al., 2018). During the school years, this can have a significant impact on academic performance and social skills, and is therefore an important area to assess to support effective learning in school (Spaniol et al., 2018).

Approximately half of the caregivers who participated in this study reported their child had difficulty in reading and writing, although codes reflecting these activities are not included in the school-aged ICF-CS for autism. The codes, *d140 Learning to read* and *d145 Learning to write*, are included, but given the extensive age range encompassed by the term ‘school-aged’, it is likely that children in the upper end of this age range will be expected to perform beyond the learning phase and competently engage in these activities. A previous study by Zajic and colleagues (2020) found writing to be an activity of particular difficulty for school-aged children on the spectrum, with the autism group demonstrating the lowest performance in text quality, word production and time spent engaged in a writing task, relative to their peers in the ADHD and non-autistic groups. Since writing is a skill often required to demonstrate academic understanding and is frequently the reason school-aged children are referred to occupational therapy services (Cartmill et al., 2009), further exploration of difficulties with this task beyond the process of learning to write may be warranted. Measures of functioning developed for this age group based purely on the school-aged ICF-CS for autism may fail to adequately capture the range of difficulties experienced by children on the spectrum during the task of writing.

### Environmental Factors

In this study, EF were more often considered facilitators than barriers. Almost all participants (99%) considered immediate family to be at least a moderate facilitator for their child, indicating that the presence of immediate family members has a predominantly positive influence on the functioning of school-aged children on the autism spectrum. Other key environmental facilitators were spread across the chapters of Products and technology (*e110*, *e115* and *e130*), Natural environment and human-made changes to environment (*e250*), Support and relationships (*e320, e330*, *e355*, and *e360*), Attitudes (*e410, e420* and e430), and Services, systems and policies (e580 and *e585*). Although it is not included in the ICF-CSs for autism, the code *e165 Assets* was reported to be at least a moderate facilitator by 79% of participants. This is unsurprising, and may need to be considered further, given the significant costs associated with obtaining a diagnosis and providing ongoing support to a child on the spectrum, with access to suitable services and supports often dictated by access to funding (Horlin et al., 2014).

Neurodevelopmental conditions including autism were previously considered using the biomedical approach and negatively viewed as an impairment to be treated or cured (Bölte et al., 2021). More recently, there has been a shift towards considering autism and other NDCs through the lens of neurodiversity, which aligns more closely with the social model of disability, suggesting the disabling impact of NDCs is a result of external barriers such as limited understanding and acceptance of neurodivergence by others (Bölte et al., 2021). The influence of the environment is underrepresented in existing measures of functioning for school-aged children on the spectrum (Hayden-Evans et al., 2022) and the ICF offers a solution for taking a more holistic and neuro-affirming approach to measuring functioning by considering the vast array of environmental factors with the potential to influence outcomes (Bölte et al., 2021).

Key environmental barriers identified included the negative impacts of sounds (*e250*) and societal attitudes (*e460*). A review by Anaby et al. (2013) also found social and societal attitudes to be significant barriers to participation for children with disabilities. Societal attitudes can be, and often are, influenced by information consumed in popular culture and entertainment. Although portrayals of autism continue to reflect stereotypes and present inaccuracies, there is evidence to suggest that this may be changing and there are more nuanced and strengths-based characters emerging in fictional media, which may positively impact societal attitudes (Jones et al., 2023). The diverse range of environmental factors considered to positively or negatively influence functioning of school-aged children on the spectrum, including physical and attitudinal factors, highlights the importance of measuring the influence of the environment on functioning (Black et al., 2022).

### Understanding of Terminology

Researchers recorded each time a caregiver requested further clarification of a code included in the ICF-NDCs to help determine which of the codes were difficult for the general population to understand. Language is one of the most important factors to consider in the development of new measures, particularly those that are self- or proxy-report, where there is unlikely to be an assessor present to answer questions or provide further explanation (Streiner et al., 2015). Understanding of codes included in the ICF-NDCs varied, with some codes requiring clarification by more than half of caregivers. This finding indicates that future iterations of the ICF-NDCs should include revised definitions of the ICF codes to ensure caregivers understand the function they are being asked to rate, and the information provided is accurate and relevant within the context.

### Limitations

In this study, only proxy-reported ratings were used to describe the functioning of school-aged children on the spectrum. The National Guideline for the Assessment and Diagnosis of Autism Spectrum Disorders in Australia recommends that information from multiple sources, including observations and consultation with other health professionals, be taken into consideration to determine a child’s level of functioning (Whitehouse et al., 2018). However, this process can be time consuming and therefore many existing measures of functioning for this population are proxy-report (Hayden-Evans et al., 2022), and used to supplement clinical information. Currently, there are limited self-report measures of functioning for school-aged children, although where possible, their active participation in the assessment process should be encouraged (Evans et al., 2022). Another limitation of this study is that the measure developed and administered, the ICF-NDCs, is scored at item-level and has no domain or total scores, meaning it is difficult to compare the results of this measure with other, established measures of functioning. As is often the case in autism research (Grebe et al., 2022), caregivers who responded in this study were primarily mothers, suggesting further research is required to determine how fathers’ perceptions of their child’s functioning may differ. Some of the children reported on in this study had co-occurring conditions, which may have impacted their functioning in the areas assessed. In future, it may be necessary to recruit two samples, with and without co-occurring conditions, to identify any influence of these co-occurring conditions on functioning. Although researchers endeavoured to recruit a representative sample, participants were conveniently sampled and thus it is possible these results do not accurately reflect the functioning of other school-aged children on the spectrum.

## Conclusions

Functioning, as a concept, is among the most difficult to define, thus measures attempting to assess functioning should be comprehensively evaluated and revised as required. The findings of this study suggest that the greatest areas of impairment or difficulty for children on the spectrum, as reported by their caregivers, largely correspond with codes included in the ICF-CSs for autism. However, some codes endorsed by more than half of participants are not currently included in the ICF-CSs for autism, or they exist only in the comprehensive ICF-CS for autism and not the age-specific set. These codes should be explored further to determine if they should be included in the ICF-CSs for autism, or added to the school-age ICF-CS for autism. Specifically, the development of future measures for school-aged children on the spectrum should consider the impact of environmental factors and draw from the broader ICF framework to include environmental facilitators and barriers relevant to other NDCs such as ADHD and CP. Some codes included in the ICF-NDCs were not clearly understood by more than half of participants, suggesting work needs to be done to review and revise the current definitions of the ICF codes prior to implementing them in self- or proxy-report measures.
